# Secretion of Hepatitis C Virus Envelope Glycoproteins Depends on Assembly of Apolipoprotein B Positive Lipoproteins

**DOI:** 10.1371/journal.pone.0004233

**Published:** 2009-01-21

**Authors:** Vinca Icard, Olivier Diaz, Caroline Scholtes, Laure Perrin-Cocon, Christophe Ramière, Ralf Bartenschlager, Francois Penin, Vincent Lotteau, Patrice André

**Affiliations:** 1 Université de Lyon, Lyon, France; 2 Inserm, U851, Lyon, France; 3 Université de Lyon1, IFR128 BioSciences Lyon-Gerland, Lyon, France; 4 Hospices Civils de Lyon, Laboratoire de Virologie Nord, Lyon, France; 5 Department of Molecular Virology, University of Heidelberg, Heidelberg, Germany; 6 CNRS, UMR 5086, Institut de Biologie et Chimie des Protéines, Lyon, France; Yale University, United States of America

## Abstract

The density of circulating hepatitis C virus (HCV) particles in the blood of chronically infected patients is very heterogeneous. The very low density of some particles has been attributed to an association of the virus with apolipoprotein B (apoB) positive and triglyceride rich lipoproteins (TRL) likely resulting in hybrid lipoproteins known as lipo-viro-particles (LVP) containing the viral envelope glycoproteins E1 and E2, capsid and viral RNA. The specific infectivity of these particles has been shown to be higher than the infectivity of particles of higher density. The nature of the association of HCV particles with lipoproteins remains elusive and the role of apolipoproteins in the synthesis and assembly of the viral particles is unknown. The human intestinal Caco-2 cell line differentiates *in vitro* into polarized and apoB secreting cells during asymmetric culture on porous filters. By using this cell culture system, cells stably expressing E1 and E2 secreted the glycoproteins into the basal culture medium after one week of differentiation concomitantly with TRL secretion. Secreted glycoproteins were only detected in apoB containing density fractions. The E1–E2 and apoB containing particles were unique complexes bearing the envelope glycoproteins at their surface since apoB could be co-immunoprecipitated with E2-specific antibodies. Envelope protein secretion was reduced by inhibiting the lipidation of apoB with an inhibitor of the microsomal triglyceride transfer protein. HCV glycoproteins were similarly secreted in association with TRL from the human liver cell line HepG2 but not by Huh-7 and Huh-7.5 hepatoma cells that proved deficient for lipoprotein assembly. These data indicate that HCV envelope glycoproteins have the intrinsic capacity to utilize apoB synthesis and lipoprotein assembly machinery even in the absence of the other HCV proteins. A model for LVP assembly is proposed.

## Introduction

Hepatitis C virus (HCV) infects an estimated 3% of the world population and frequently causes chronic infection often leading to cirrhosis and liver cancer. The virus was first isolated in 1989 by molecular biology techniques [1] and classified in the Hepacivirus genus within the *Flaviviridae* family, which includes the flaviviruses (e.g. yellow fever virus and Dengue virus), the pestiviruses (e.g., bovine viral diarrhoea virus), and GB viruses [2]. However, the high frequency of chronic infections and the very narrow host range limited to humans and chimpanzees sets HCV apart from the other flaviviruses. Since its discovery, many aspects of the HCV replication cycle as well as the pathophysiology of chronic hepatitis C have been described (for recent reviews, see [3], [4]). Surprisingly, despite the possibility to propagate the virus *in vitro*, the precise nature of the HCV particle is still poorly understood in contrast to the flaviviruses that have been studied in great details [5]–[7]. Unique to HCV is the exceptional low density of the virus particles resulting from the association of the virus with lipoproteins [8], [9]. Low density complexes of HCV and lipoproteins are preferably observed in the blood of chronically infected patients while most of the viral particles produced *in vitro* have a density similar to that of flaviviruses [10], [11].

The density of the blood circulating forms of HCV is very heterogeneous ranging from 1.25 to less than 1.06 g/mL. Particles with high density could correspond to naked capsids [12]. Particles in plasma density fraction around 1.15 g/mL may represent conventional viruses similar to those produced in Huh-7 cells that are derived from the highly replication competent JFH1 strain (HCVcc) [13]–[15]. Viral particles in density fractions below 1.06 g/mL are associated with apolipoprotein B (apoB) bearing triglyceride rich lipoproteins (TRL), namely the low, intermediate and very low density lipoproteins (LDL, IDL and VLDL, respectively) and chylomicrons [9], [10], [16]–[19]. This uncommon association of a virus with lipoproteins is of particular interest since viral particles of low density have a higher specific infectivity than high density particles, *in vivo* for chimpanzees and *in vitro* in the Huh-7 cell culture system [11], [20], [21]. A transmission case of hepatitis C suggests that low density viral particles are also infectious in humans [22]. It is not clear however, whether every circulating HCV particles are associated with apoB, the triglyceride content of the particle being the parameter changing the density, or whether only the low density particles are apoB positive and triglyceride rich viral complexes.

Because of their association with TRL, the low density particles have been assigned the name of lipo-viro-particles (LVP) [10]. The proportion of LVP amongst the circulating viral particles varies from patient to patient, but on average almost half of HCV RNA is detected in the circulating plasma fractions with density lower than 1.06 g/mL. LVPs are recognized by host antibodies and these immunoglobulin positive particles can be purified by protein A precipitation. Electron microscopy studies identified purified LVPs as globular particles that are heterogeneous in size with an average diameter of 100 nm. These contain higher amounts of triglycerides than lipoproteins isolated from the same density fractions and they contain apolipoproteins (B, CII, CIII, and E, but not the HDL-associated apoA) as well as the viral RNA, core protein and envelope glycoproteins E1 and E2 [10], [18]. Treatment of LVP with detergent does not destroy the association of HCV RNA with apoB [18]. Surprisingly, the two apoB isoforms, apoB 100 and apoB 48, are present in LVP with comparatively more apoB 48 in LVP than in the plasma [16]. While apoB 100 is produced by the liver, apoB 48 is only synthesized by enterocytes and is essential for the formation of chylomicrons [23]. This is in keeping with the existence of an intestinal site of HCV assembly and maturation that is supported by the detection of viral non structural proteins in enterocytes of chronically infected patients and the change in neutral lipid composition of LVP content early after a fat rich meal [16], [24].

The nature of LVP however remains poorly defined and the process leading to the coassembly of lipoproteins and virus hybrid complexes is not understood. It has been suggested that LVP formation occurs at the ER membrane where TRL synthesis takes place [25] since HCV RNA can already be immunoprecipitated by anti-apoB antibodies in chronically infected liver macerates [26]. In support to this hypothesis, it was recently shown that *in vitro* production of HCV in the Huh-7 cell line depends on the assembly of VLDL and on the expression of apoE [27]–[29]. It has also been documented that HCV envelope glycoproteins E1 and E2 are retained in the endoplasmic reticulum (ER) by retention signals in their transmembrane domains (for review see [30], [31]). Concerning LVP, E1 and E2 appear to be exposed on the surface of purified LVP since they can be recognized by anti-envelope antibodies under non denaturating conditions [16]. The envelope glycoproteins may thus play a pivotal role in the formation of LVP. To better understand the interaction between envelope glycoproteins and TRL and the assembly of LVP, E1 and E2 proteins were stably expressed in cell lines secreting apoB positive lipoproteins. It is shown here that the viral glycoproteins are secreted from these cells only when the TRL synthesis and secretion pathway is functional. Moreover, the resulting hybrid TRL particles bear E1 and E2 glycoproteins at their surface. These findings reveal that HCV envelope glycoproteins have the intrinsic capacity to assemble into TRL.

## Results

### Establishment and characterization of Caco-2 cells expressing HCV envelope glycoproteins

**Figure 1:**
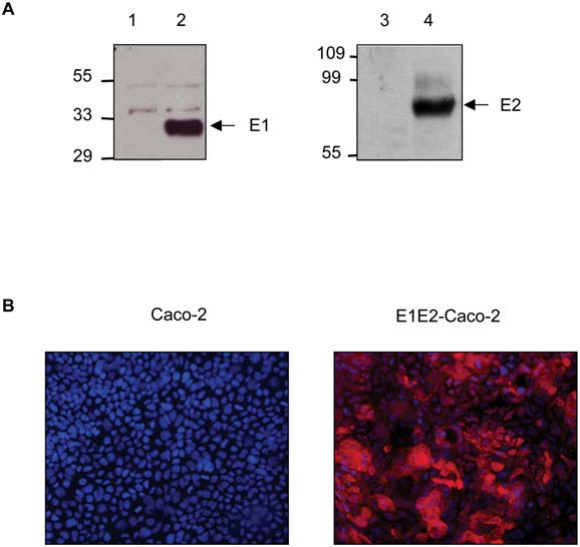
Expression of the HCV glycoproteins E1 and E2 in Caco-2 cells. Caco-2 cells maintained under standard culture conditions after transduction with the E1E2-HIV-SIN lentiviral vector express E1 and E2 proteins. (A) Western blot analysis showing E1 and E2 revealed as single bands by anti-E1 antibody, (lane 2), and anti-E2 antibody (lane 4), at their expected molecular weight in transduced cells, but not in control cells (lanes 1 and 3, respectively). (B) Expression of envelope glycoprotein remains stable. Staining of transduced cells with anti-E2 antibody (in red) shows that almost all cells still expressed the envelope protein after at least 40 passages.

**Figure 2:**
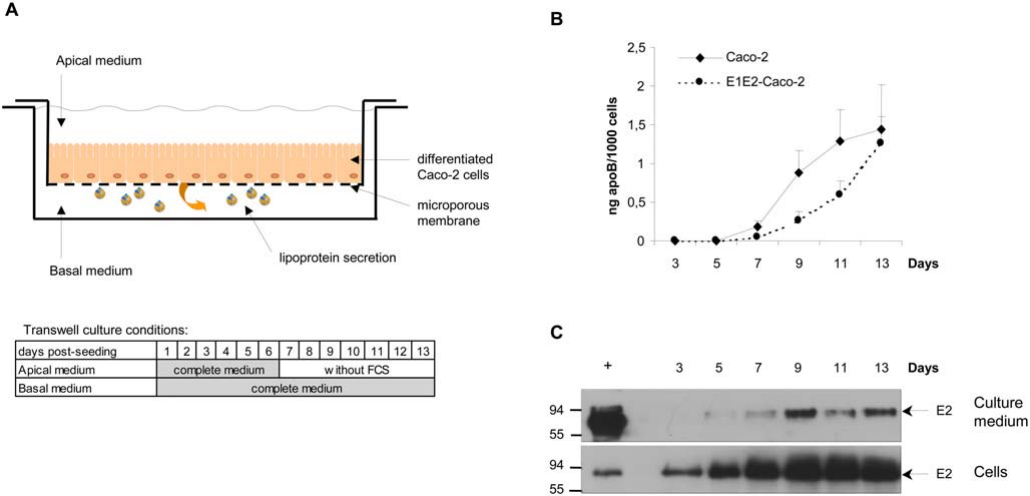
Kinetic of ApoB and HCV glycoprotein secretion during Caco-2 cell differentiation. (A) Caco-2 cells were grown on porous filters under asymmetric medium conditions as depicted for two weeks after seeding. Media in upper and lower chambers were changed every two days. Under these differentiation conditions, basolateral lipoprotein secretion begins after one week of culture [32]. (B) ApoB secreted by control and E1E2 transduced cells into the basal medium was measured by ELISA. Values indicate apoB (ng) secreted by one thousand cells for two days per mL of culture medium. Vertical bars indicate standard deviations. The kinetic is representative of three separate experiments. (C) Expression and secretion of E2 protein by E1E2-Caco-2 cells during differentiation. Content of E2 protein in 40 µl of basal medium, upper pannel, and, in cell lysate, lower panel, (per 20 µg total protein), was analysed every two days by Western blotting with the E2-specific H52 antibody. As indicated in Figure 1, E2 was detectable in seeded cells and remained present during the two weeks of culture. Its expression increased during the early phase of the differentiation until day 9 when a plateau was reached. E2 secretion into the basal medium began at day 7, concomitantly with apoB secretion. Secreted E2 appeared to have a higher molecular weight than the control intracellular E2 protein likely because of a higher glycosylated state.

The human intestinal cell line Caco-2 does not synthesize apoB and MTP when cultured under standard conditions and therefore does not secrete apoB-containing lipoproteins. Caco-2 cells maintained under these conditions were infected with the E1E2-HIV-SIN lentiviral vector. After transduction, E1 and E2 proteins with the expected molecular weight could be detected in cell lysates by Western blotting (Figure 1A). Immunofluorescence analysis indicated that almost all cells expressed the glycoproteins (Figure 1B) and that the expression was stable for at least 40 passages. However, when cultured on porous filter allowing asymmetrical culture conditions with serum free medium in the upper chamber and complete medium in the lower chamber of the culture device, Caco-2 cells partially differentiate into polarized cells and regain the capacity to synthesize and secrete TRL at their basal membrane into the lower chamber a few days after cells reach confluence (Figure 2A) [32], [33].

As shown in the time course experiment in Figure 2B and in agreement with previous reports [32], [33], apoB could only be detected in the basal compartment of the culture 7 days after cell seeding. Concentrations of apoB then increased until day 13. Interestingly, in E1E2 expressing cells, the apoB secretion was significantly delayed but still reached a similar high level at day 13. Although E2 was always detected in cell lysates (Figure 2C), a fraction of E2 was detected in the basal medium concomitant with the apoB secretion, suggesting that HCV envelope proteins are secreted with apoB. Cell differentiation was also correlated with an increased amount of intracellular E2 (Figure 2C).

### HCV glycoproteins are not secreted by the apoB negative epithelial Hela cell line

**Figure 3:**
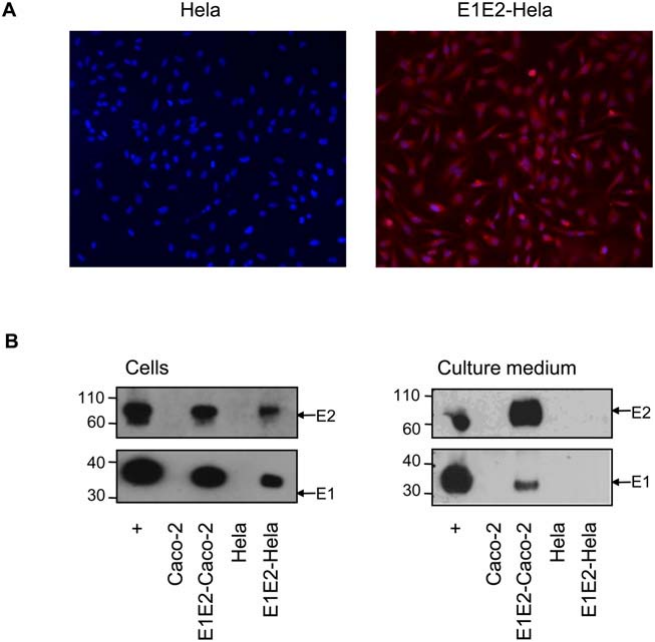
Epithelial Hela cells do not secrete HCV glycoproteins. (A) Analysis of HCV glycoprotein expression by E1E2-Hela by immunofluorescence with E2-specific antibody H52. Cells were grown under standard conditions and stained as described in materials and methods. Left panel, control cells, right panel, E1E2-Hela. E2 positive cells are stained in red. (B) Caco-2 and E1E2-Caco-2 cells were cultured under differentiating conditions for 11 days followed by 2 days in FCS free medium before baso-lateral medium and cells were collected. Hela and E1E2-Hela cells were cultured in standard conditions until 80% confluence and then kept for two days in FCS free medium before cells and supernatants were collected. Cellular and medium samples for western blotting were adjusted for the total cellular protein contents. HCV glycoproteins were detected in both E1E2-Caco-2 and E1E2-Hela cell and not in control cells (left panel) but the two glycoproteins were only secreted by E1E2-Caco-2 and not by E1E2-Hela and control cells (right panel).

The secretion of HCV glycoproteins was only detected concomitantly with the synthesis and secretion of apoB positive lipoproteins by E1E2-Caco-2 cells suggesting that a functional apoB pathway is necessary for E1E2 secretion. We then tested if the two glycoproteins could nevertheless be also secreted by epithelial cell line not from hepatic or intestine origin and not producing lipoproteins. Hela cells were transduced by the E1E2 lentiviral vector and were found, as Caco-2 cells, to stably express the glycoproteins as shown by immunostaining (Figure 3A). By contrast, the glycoproteins were not detected in the supernatant of E1E2-Hela cells as they were in E1E2-Caco-2 basal medium (Figure 3B).

### Characterization of the apoB and HCV envelope glycoprotein complexes

**Figure 4:**
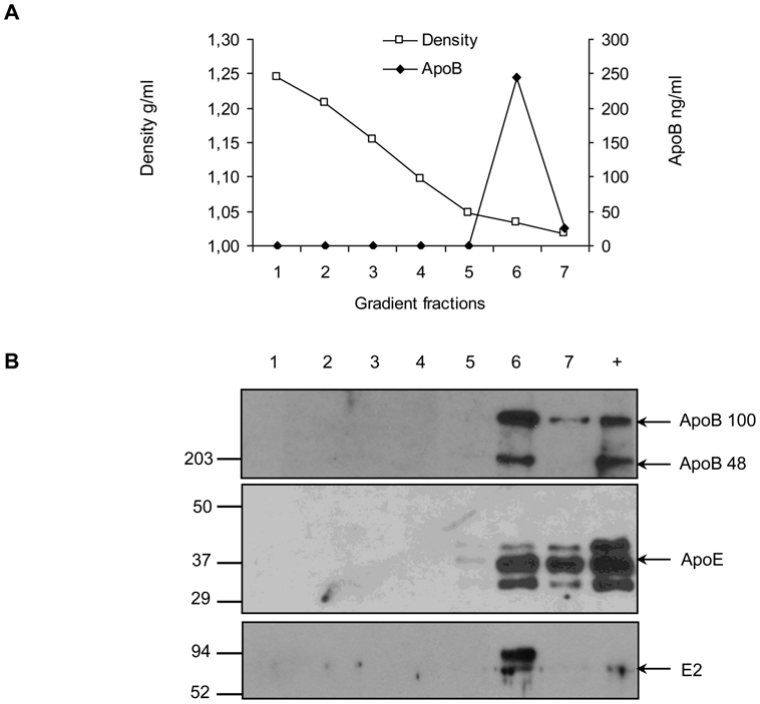
Density and nature of apolipoproteins and HCV glycoproteins secreted into Caco-2 basal medium. E1E2-Caco-2 cells were grown on porous filters under asymmetric medium conditions for 11 days. Upper and lower culture medium were then changed and replaced with foetal calf serum free medium. Cells were incubated for two additional days. Basal medium was collected and run on an iodixanol gradient. (A) Density fractions were collected and apoB concentrations per mL of basal medium were determined by ELISA and expressed in ng/ml. ApoB was only present in fractions with density ≤1.05 g/mL. (B) Density fractions were analysed by western blotting using specific antibodies against apoB, apoE and E2 glycoprotein. ApoB positive fractions contained the two apoB isoforms, apoB100 and apoB48. ApoE and E2 were only detected in the apoB positive fractions. Control proteins were deposited in lane +.

**Figure 5:**
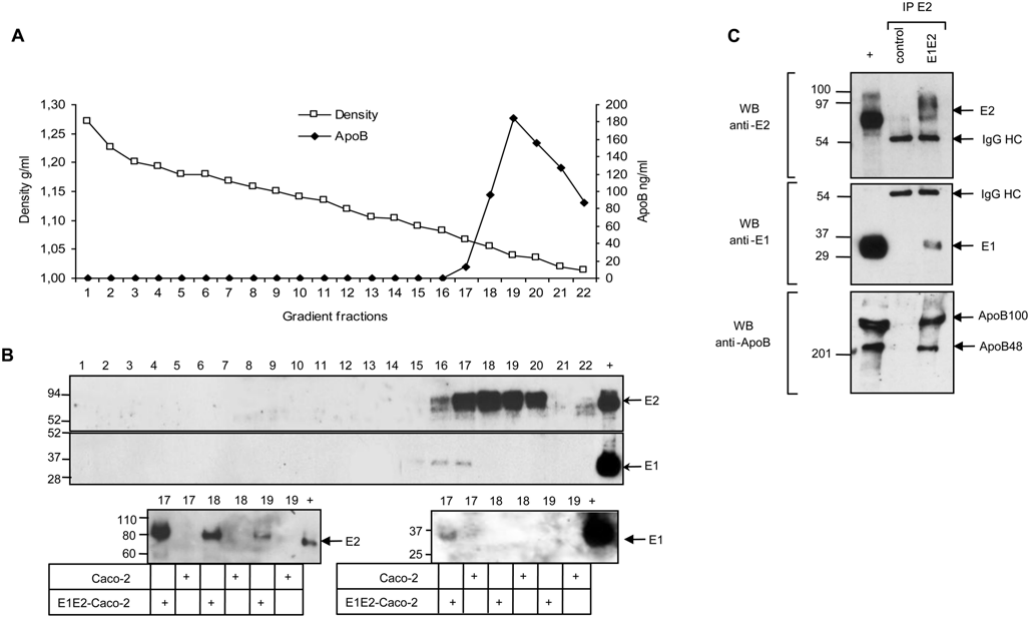
Characterization of the apoB and envelope containing particles secreted into Caco-2 basal medium. Cells were cultured as in Figure 4. Basal medium was collected at day 13 after two day incubation in serum free medium. Density of apoB and E1E2 glycoprotein positive fractions in the E1E2-Caco-2 basal medium. Basal medium was run on a iodixanol gradient and fractions were analysed by apoB Elisa (A) and western blotting (B, upper panel) stained with envelope specific H52 and A4 antibodies. E1 and E2 proteins were present in apoB positive fractions with the higher density leaving the upper apoB fractions free of envelope glycoproteins. E1 was only detected in the fractions with the highest density. Intracellular E1 and E2 control proteins were deposited in the last right lane (+). To further assess the specificity of the antibody, fractions obtained from E1E2-Caco-2 and control Caco-2 grown in the same conditions were normalized for apoB concentrations and ran in parallel in one western blot (B, lower panel). Envelope glycoproteins were only detected in fractions obtained from E1E2-Caco-2. (C) Immuno-precipitation of E2 positive particles. Basal culture medium of control Caco-2 and E1E2-Caco-2 cells were collected at day 13 of differentiation after two days of culture in serum free medium. E2 positive particles were captured by E2-specific H48 monoclonal antibody and immuno-precipitated particles were analysed by western blotting and stained with anti-E2 H52 (upper panel), anti-E1 A4 (middle panel) and anti-apoB (lower panel) antibodies as described in materials and methods section. Molecular weights are indicated on the left, control proteins were deposited in the + lane. Identical volume of precipitated material either from E1E2-Caco-2 or control Caco-2 basal medium were deposited in lanes “E1E2” and “control” respectively for every western blot.

To further investigate the association of glycoproteins and TRL, E1E2 expressing and control Caco-2 cells were cultured under differentiation conditions and the basal medium collected at day 13 was analyzed in an iodixanol density gradient. Figure 4 shows that the glycoprotein E2 and isoforms of apoB and apoE were detected in fractions with the lowest density (≤1.05 g/mL) with the maximum concentration in the 1.035 g/mL fraction. Concerning apoB, the two isoforms, apoB100 and apoB48, were secreted in comparable molar concentrations. This further confirms the differentiation state of the cells that can edit, at least partially, the apoB mRNA to synthetize apoB48 [32]. The distribution of the two glycoproteins E1 and E2 in a highly resolved density gradient is shown in Figure 5. Both glycoproteins co-sedimented in apoB containing fractions from E1E2-Caco-2 cells but not in corresponding fractions from control Caco-2 cells (Figure 5 A and B). Interestingly, although apoB was still present in fractions with the lowest density of about 1.02 g/mL, the two glycoproteins and particularly E1 were confined to the apoB positive fractions with a density from 1.07 to 1.05 g/mL suggesting that different lipoproteins were secreted into the basal medium; one group of very light lipoproteins that are not associated with the glycoproteins and the group of hybrid lipoproteins which had a higher density presumably because of the additional viral proteins. E1 was mainly detected as monomer (apparent MW of ∼30 kD) in the fractions with the highest density (fraction 16 and 17) and not in the other fractions where E2 was also detected. Some additional bands with molecular weight of 70 kD that may correspond to E1 trimers [34] were also detected in the other E2 positive fractions (data not shown). However, these high molecular weight bands co-migrated with a non specific cross reacting protein present in apoB positive fractions leaving open the question of the existence of E1 trimers associated with apoB particles of lower density. Alternatively, the weakest reactivity of anti-E1 antibody on western blot than the anti-E2 antibody reactivity might limit the detection of E1. Finally both glycoproteins might not be secreted with the same efficiency.

To confirm that both glycoproteins are associated with lipoproteins, complexes from unfractionnated basal medium were immuno-precipitated using an E2-specific conformational monoclonal antibody. Only immuno-precipitated complexes from E1E2-Caco-2 cells but not from control cell basal medium (Figure 5C) contained E1 and the two isoforms of apoB in addition to E2. Taken together these data indicate that E1 and E2 glycoproteins can be integrated into at least some TRL that are secreted at the basolateral side of differentiated epithelial cells and that they are present on the surface of the lipoprotein complexes.

### HepG2 but not Huh-7 cell lines secretes HCV glycoproteins as hybrid TRL

**Figure 6:**
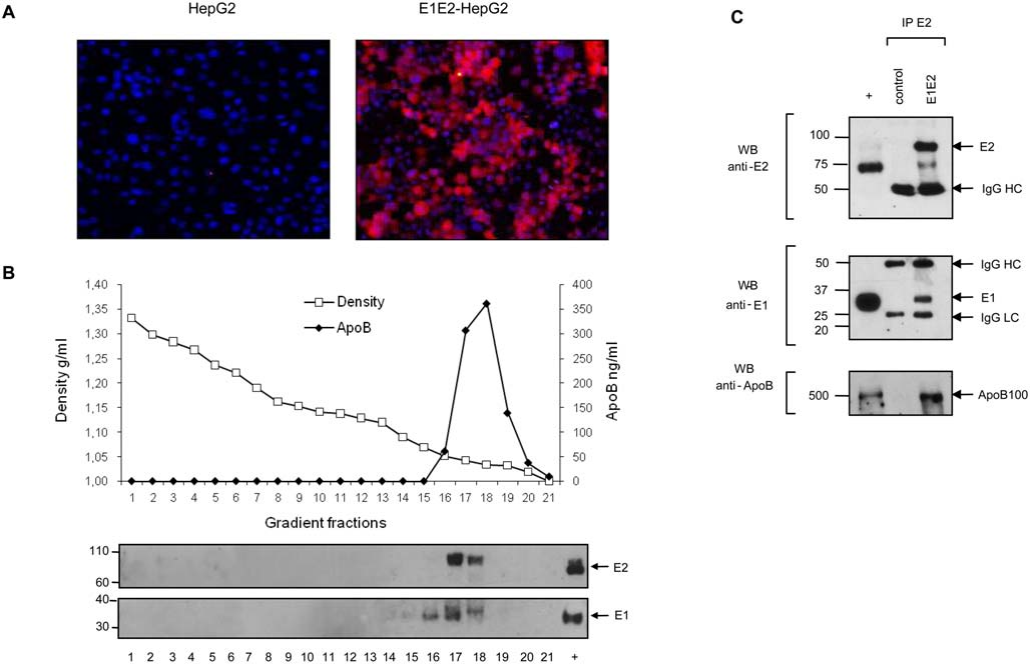
Characterization of particles secreted by HepG2. (A) Analysis of HCV glycoprotein expression by immunofluorescence with E2-specific antibody H52. HepG2 cells were grown under standard conditions and stained as described in materials and methods. Left panel, control cells, right panel, E1E2 transduced HepG2 cells. E2 positive cells are stained in red. (B) Density of apoB and E1E2 positive fractions in the E1E2-HepG2 supernatant. Three day culture supernatant was run on an iodixanol gradient and fractions were analysed by apoB ELISA, upper panel, and western blotting with E2-specific antibody, lower panel. Envelope control proteins were deposited in the last right lane (+). E2 proteins were only present in apoB positive fractions. (C) Immuno-precipitation of E2 positive particles was performed as in figure 5 with HepG2 supernatant collected three days after seeding. Basal culture medium of control and E1E2-HepG2 were collected after two days of culture in serum free medium. E2 positive particles were captured by E2-specific H48 monoclonal antibody and immuno-precipitated particles were analysed by western blotting and stained with E2-specific H52 antibody (upper panel), E1-specific A4 antibody (middle panel) and apoB-specific antibody (lower panel). Molecular weight are indicated on the left, control proteins were deposited in the + lane.

To extend the results found with intestinal cells to liver cells, and as HCV predominantly replicates in the liver, the viral glycoproteins were expressed by transduction with the lentiviral particles in the human hepatic cell line HepG2, often used as a model for TRL secretion. As observed with the Caco-2 model, E1 and E2 secreted in the supernatant were only found in low density fractions with apoB (Figure 6B). Similarly to the Caco-2 model, the E1E2-HepG2 cells secreted the glycoproteins as hybrid apoB positive particles, which could be immuno-precipitated with the E2-specific monoclonal antibody (Figure 6C). However, as expected, apoB was only found as the apoB100 isoform since human liver cells cannot edit the apoB mRNA.

**Figure 7:**
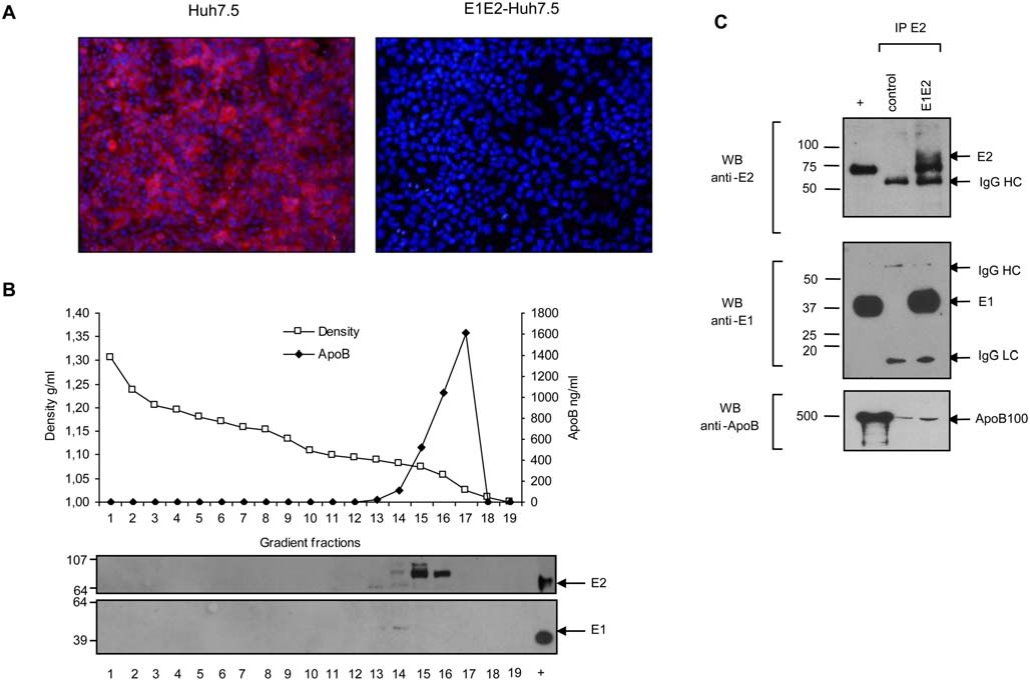
Characterization of particles secreted by Huh-7.5 cells. (A) Analysis of HCV glycoprotein expression by immunofluorescence with E2-specific antibody. Huh-7.5 were grown under standard conditions and stained as described in materials and methods. Left panel, control cells, right panel, E1E2 transduced Huh-7. E2 positive cells are stained in red. (B) Density of apoB and E1E2 positive fractions in the E1E2-Huh-7.5 supernatant. Three day culture supernatant was run on a iodixanol gradient and fractions were analysed by apoB ELISA, upper panel, and western blotting with E1 and E2-specific antibodies, lower panel. Envelope control proteins were deposited in the last right lane (+). E1 and E2 proteins were only present in apoB positive fractions. (C) Immuno-precipitation of E2 positive particles was performed as in figure 5 with Huh-7.5 supernatant collected three days after seeding. Basal culture medium of control and E1E2-Huh-7.5 were collected after two days of culture in serum free medium. E2 positive particles were captured by E2-specific H48 monoclonal antibody and immuno-precipitated particles were analysed by western blotting and stained with H52 anti-E2 (upper panel), A4 anti-E1 (middle panel) and anti-apoB (lower panel) antibodies. Molecular weight are indicated on the left, control proteins were deposited in the + lane.

**Figure 8:**
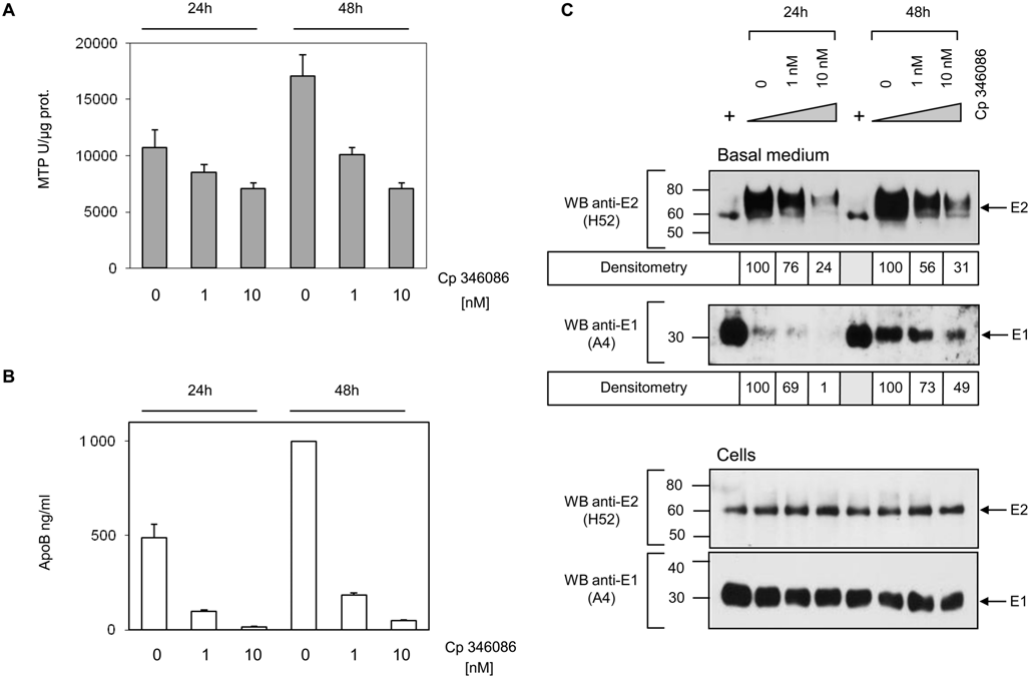
MTP activity inhibition decreases HCV envelope glycoprotein secretion by E1E2-Caco-2. E1E2-Caco-2 cells were cultured under differentiating condition for 11 days when medium was replaced by FCS free medium with the MTP inhibitor Cp 346084 at 0, 1 or 10 nM. MTP activity was measured on frozen lysates and expressed as described in the Materials and Methods section. (A) MTP activity in lysate of E1E2-Caco-2, 24 and 48 h after addition of Cp 346084. MTP activity was not completely abrogated by the addition of the inhibitor since its activity continued to increase during the inhibition period. However, there was a dose dependant inhibition of MTP activity by 1 and 10 nM Cp 346084. (B) Concentrations of secreted ApoB in the basal medium were measured by ELISA at 24 and 48 h after addition of 1 or 10 nM Cp 346084. ApoB secretion was inhibited in a dose dependant manner by the MTP inhibitor. (C) Secretion of HCV glycoprotein E1 and E2 in the basal medium was monitored by western-blotting with the E1-specific A4 and E2-specific H52 antibodies at 24 and 48 h after addition of 1 or 10 nM Cp 346084. Control cellular E2 protein was deposited in lanes (+). Amount of secreted E1 and E2 was quantified by densitometry as per cent of secreted glycoprotein in absence of inhibitor (upper panels). Cellular glycoprotein expression was monitored by western-blotting of cell lysates and was not modified by MTP inhibition (lower panel).

When testing E1E2-Huh-7.5 cells that support HCVcc replication, we also found that E1 and E2 were secreted into low density fractions of the supernatant (Figure 7B) indicating that these proteins were associated with lipids. But in strong contrast with the Caco-2 and HepG2 models and despite many attempts, if it was still possible to co-immunoprecipitate E1 with E2-specific antibody, it was not possible to co-immunoprecipitate apoB and, in the best cases as shown on Figure 8C, only traces of apoB, and not much more than from control cell supernatant, could be detected on these E1 and E2 positive low density complexes. We obtained the same lack of association of envelope glycoproteins and apoB with a parental Huh-7 cell line (data not shown). These data suggest that in Huh-7 cell lines, apoB positive lipoproteins and lipid associated envelope glycoproteins could likely not fuse in single hybrid particles.

### Inhibition of MTP decreases apoB and viral glycoproteins secretion

MTP (microsomal triglyceride transfer protein) is an essential protein in the synthesis and assembly of TRL in both the liver and intestine. To evaluate the role of MTP in the synthesis and assembly of the hybrid glycoproteins-TRL particles, Caco-2 cells were grown under conditions of differentiation for 11 days before the MTP inhibitor Cp 346086 was added [35]. MTP activity and the secretion of apoB and E2 were measured one and two days later. As shown in Figure 8A, MTP activity was inhibited in a dose dependant manner by 35 and 50% at one or two days after addition of the inhibitor, respectively. ApoB secretion was reduced in parallel in similar proportions (Figure 8B) while the secretion of MIP-1 beta in the basal medium was not affected suggesting that the MTP inhibitor specifically reduced the apoB lipoprotein secretion but not the secretion of unrelated proteins (data not shown). Most importantly if the intracellular expression of the two glycoproteins was not modified by the MTP inhibition, the quantity of E1 and E2 secreted into the basal medium after 24 h or 48 h incubation with 1 and 10 nM inhibitor, was greatly reduced and correlated with the concentration of secreted apoB (Figure 8C).

**Figure 9:**
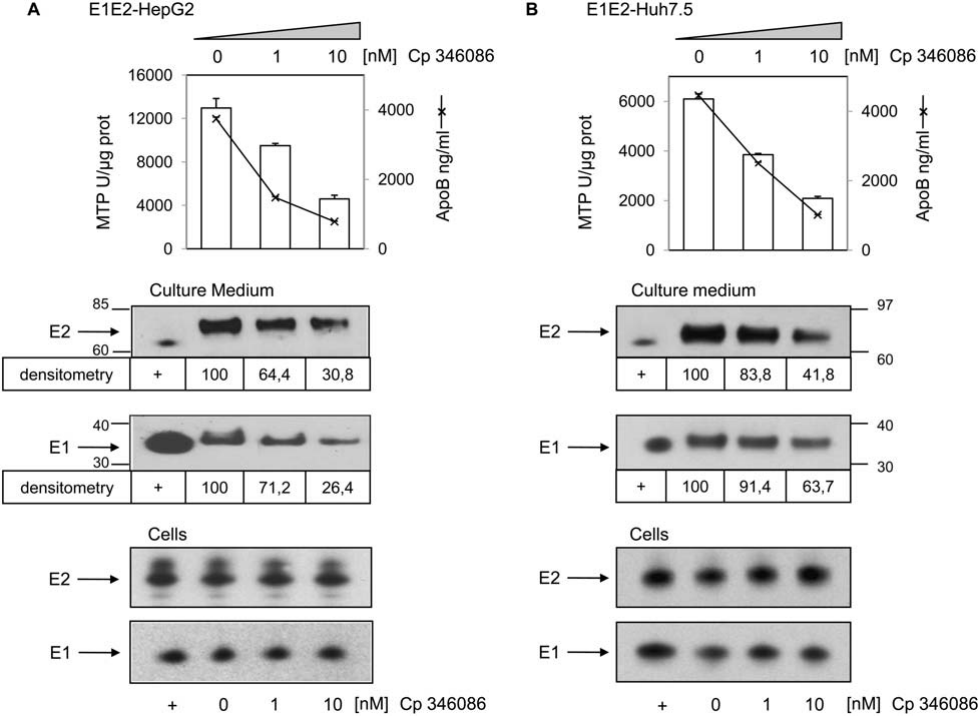
MTP activity inhibition decreases HCV envelope glycoprotein secretion by E1E2-HepG2 and E1E2-Huh-7.5. E1E2-HepG2 and E1E2-Huh-7.5 cells were cultured in standard conditions until 80% confluence when medium was replaced by FCS free medium with 0, 1 or 10 nM of MTP inhibitor Cp 346084 for two days. MTP activity was measured on frozen lysates and expressed as described in the Materials and Methods section. (A) E1E2-HepG2 and (B) E1E2-Huh-7.5. Upper panels, MTP activity in cells and apoB concentrations in medium in presence of the indicated inhibitor concentrations. Middle panels, quantification of secreted glycoproteins in presence of 1 and 10 nM MTP inhibitor by western blotting and densitometry as per cent of secreted glycoproteins in absence of inhibitor. Lower panels, cellular glycoprotein expression was not modified by the MTP inhibitor as shown by western-blotting of cell lysates.

The effect of MTP inhibition on the two glycoproteins secretion by E1E2-HepG2 cells was very similar to that observed with E1E2-Caco-2 cells (Figure 9A) with around 40% reduction of E2 secretion and 30% reduction of E1 secretion after 48 h in presence of 1 nM inhibitor. The same effect was also observed with E1E2-Huh-7.5 cells (Figure 9B) but to a lower extent with only 16% and 8% reduction of respectively E2 and E1 secretion in the same inhibitor conditions. The effect of MTP inhibition on the two glycoproteins secretion seemed also to depend on the lipoprotein pathway since the secretion of albumin by these two liver derived cell lines was not modified (data not shown).

## Discussion

In both Caco-2 and HepG2 cell culture systems, the HCV glycoproteins are secreted with the apoB positive lipoproteins. This secretion is strictly dependent on an efficient apoB positive lipoprotein secretion pathway because, first, apoB negative epithelial Hela cells do not secrete HCV envelope proteins, second, Caco-2 cells only secrete the viral glycoproteins after having acquired the apoB synthesis and secretion capacity and, finally, inhibition of a normal lipidation of apoB with an MTP inhibitor reduced the amount of the secreted viral glycoproteins. The E1 and E2 envelope proteins thus contain the signals mandatory to interfere with a functional apoB pathway in liver and intestinal cells. It is worth noting that both systems seem to be interdependent. Indeed, the amount of cellular viral glycoproteins increases accordingly to apoB and conversely the presence of E1 and E2 likely delays the secretion of apoB. A metabolic syndrome frequently associated with hepatitis C includes insulin-resistance, liver steatosis and hypo-beta-lipoproteinemia. Decreased serum apoB concentrations have been attributed to inhibition of MTP activity by HCV core protein [36], [37] and by the facilitated degradation of apoB by the non structural protein NS5A [38] which both could reduce the production of TRL. Here we found that expression of E1 and E2 delays apoB secretion by Caco-2 cells providing another mechanism for reduced apoB serum concentration.

**Figure 10:**
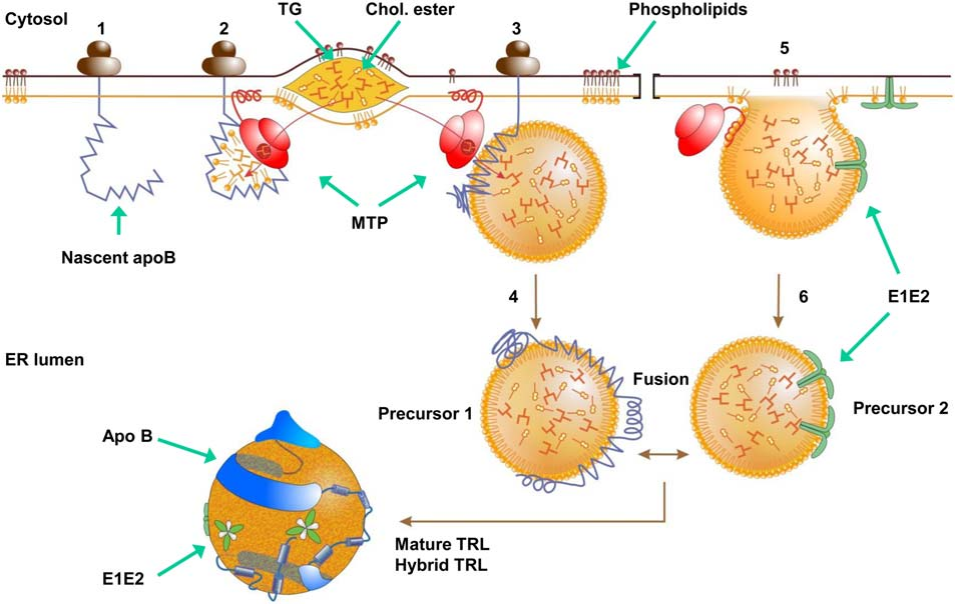
Model of TRL assembly and formation of HCV envelope glycoprotein hybrid TRL (adapted from Shelness [41]). During its translation at the rough ER, the nascent apoB peptide is translocated within the ER lumen (step 1) where a lipid cavity is formed. This lipid cavity is closed by MTP allowing the recruitment of a small amount of phospholipids and triglycerides (TG) (step 2). Continued apoB translation and MTP-mediated TG transfer form a neutral lipid core and conversion to a spheric particle (step 3) that is then released into the ER lumen after translation completion. This first precursor then moves to a distal compartment of the secretory pathway (step 4). In the smooth ER, a second precursor is formed from the ER membrane and MTP-mediated TG enrichment (step 5) and then traffics to the secretory pathway (step 6) where the two precursors meet and fuse to form mature TRL (fusion step). Several factors are required for the formation of the second precursor and the fusion between the two precursors (see text). HCV envelope glycoproteins are retained in the ER membrane after their maturation and clivages by ER peptidases. We hypothesise that E1E2 dimers might diffuse from the ER membrane constituted of two phospholipid monolayers to the budding second precursor. Transition from a two leaflets membrane to the phospholipid monolayer membrane of the precursor might induce conformation changes favouring the formation of E1E2 trimers triggered by the trimerisation of E1 as recently proposed [34]. The glycoprotein loaded second precursor can then fuse with the first precursor leading to hybrid TRL as observed in Caco-2 and HepG2 but not in Huh-7 cells (see text).

Transduction of the gene coding for E1 and E2 without any additional viral genes is sufficient to induce the assembly and the secretion of low density hybrid particles resembling TRL and bearing the viral glycoproteins on their surface. The current VLDL or chylomicron assembly model is envisioned as a two steps process which requires at least apoB and MTP (for reviews see [39]–[41] and Figure 10). During the first step, apoB is synthesized and translocated into the rough ER lumen forming a lipid binding cavity that is completed with MTP. MTP thus allows the recruitment of phospholipids and triglycerides (step 2). The ongoing translation of apoB concomitant with MTP mediated lipid transfer from the ER lipid droplets forms a neutral lipid core and conversion to a spherical emulsion particle (step 3). After release from the ribosome, the apoB precursors (precursor 1) move to a distal compartment in the secretory pathway (step 4) where they meet with apoB-free luminal lipid droplets or second precursor, formed by MTP in the smooth ER (step 5–6). The first and second precursors fuse to form mature TRL, a process that may be facilitated by apoE, another major component of TRL [42], [43]. In that model, it seems unlikely that a transfer of the viral glycoproteins from the ER membrane where they reside to the apoB precursor may occur. Indeed such a leap would need energy and/or complex tertiary or quaternary rearrangement of the glycoproteins induced by an hypothetical binding to the nascent precursor and allowing the transmembrane domain to detach from the ER membrane. The formation of the apoB free precursor is less well understood (step 5–6). If it also depends on MTP, the mechanism leading to a luminal lipid droplet limited by a monolayer of phospholipids is not known. It is tempting to hypothesize that E1 and E2 may slide from the ER membrane to the budding apoB free precursor without any energetically costly conformational changes. In Caco-2 and HepG2 cells, the envelope proteins associated with the second precursor would then fuse with the apoB precursors to form mature hybrid particles. On the contrary, in Huh-7, the fusion likely does not occur and envelope proteins are secreted associated with lipids, and likely with the second precursors, a process that seems to depend on MTP since the secretion of both glycoproteins could be reduced by an MTP inhibitor but less efficiently than in Caco-2 and HepG2 cells. E1 and E2 might therefore be envisioned as markers of the second precursor supporting the two precursor model of lipoprotein synthesis.

These data support the recently published hypothesis that some viral particles could result from co-assembly of viral particles and lipoproteins [25] and at least partially explain why apoB and/or MTP inhibition reduces the production of infectious viral particles in Huh-7 cells [28], [29]. Chang and colleagues in addition showed that ApoE is required for the production of infectious virus in cell culture [27]. As apoE is involved directly or indirectly in the second precursor formation [43], the reduction of intracellular and secreted virions by apoE knockdown could result from the suppression of the second VLDL precursor assembly. Altogether, these data indicate that both steps of VLDL formation and secretion are needed for efficient virion production and suggest that secreted virions are associated with apoB while other particles are degraded [28]. Interestingly however the latter data were obtained in Huh-7 that is shown here to be defective for proper assembly of VLDL with a defect either in the second precursor formation or in the two precursor fusion step since apoB and glycoproteins are present on separate low density molecular complexes. Virions produced in Huh-7 might benefit from the second precursor to assemble and to be associated with lipid in an MTP dependent manner, but could only marginally fused with the apoB precursor leading to particles with some density heterogeneity that cannot be immunoprecipitated by anti-apoB antibody [29]. The pathways for biosynthesis of TRL triglyceride both in liver and intestine involve several key enzymes [44], [45] which may not be fully functional in Huh-7.

In our experimental system, the formation of the hybrid lipoproteins only required the presence of the envelope glycoproteins. Conversely, the formation of infectious virions may benefit from the budding of the hybrid precursor. Recently, it was proposed that HCV core localizes on the monolayer membrane of cytosolic lipid droplets and then recruits replication complexes associated with the ER membrane forming a membranous local environment critical for producing infectious viruses [46], [47]. It is thus tempting to speculate that, in this local environment, assembling capsids might profit of the budding hybrid second precursor driven by the envelope glycoproteins and MTP to translocate within the precursor lipid core to form an uncompleted viral particle. That particle could secondly fuse in competent cells with the first precursor in the presence of apoE forming the lipo-viro-particles. Translocation of the nucleocapsid limited by the core hydrophobic domains into the lipid core may be energetically favored by the phospholipid monolayer surrounding the hybrid precursor.It is likely that many defective particles circulate in the blood of chronically infected patients since particles containing only E1 and E2 can be produced in large amount as observed in this system. Previous LVP studies reported that purified LVP contained more apoB than HCV RNA molecules suggesting that some hybrid particles recognized by endogenous antibody are defective viral particles [10], [16].

This study shows that viral envelope proteins interfere with the apolipoprotein B pathway to be secreted and provides further evidences on the nature and on the assembly of LVP. Hepatitis C virus also appears as a valuable tool for studying the successive steps of VLDL and chylomicron synthesis and secretion.

## Materials and Methods

### Materials

Unless indicated, all chemicals were from Sigma. Monoclonal antibodies anti-E1 (clone A4) or E2 (clones H52 and H48) mAbs were obtained from Dr J. Dubuisson (Institut de Biologie de Lille-Institut Pasteur de Lille, France). Anti-apoB (clone 1D1) mAb was from the Heart Institute (University of Ottawa, Ontario, Canada), anti-apoB (clone 1609) monoclonal antibodies and peroxidase-conjugated goat anti-apoB antibodies were from Biodesign (Saco, ME, USA). Anti-apoE (clone 3D12) was obtained from Millipore (Molsheim, France). The MTP inhibitor CP346086 [35] was kindly provided by G. Luo (Department of Microbiology,University of Kentucky College of Medicine, Lexington, Kentucky). Cell culture reagents were obtained from Invitrogen (Cergy-Pontoise, France) and FCS (foetal calf serum) from PAN-Biotech GmbH (Aidenbach, Germany). Microporous polyethylene terephthalate membrane inserts were from Becton Dickinson (Le Pont de Claix, France) (4.2 or 0.3 cm^2^, 1 µm pore size).

### Lentiviral particles production and cell transduction

A lentiviral vector E1E2-HIV-SIN for the transduction of the HCV glycoproteins E1 and E2 was constructed. The gene coding the E1 and E2 from HCV genotype 1a H77 strain (AJ 318514, nucleotides 733 to 2579) were introduced at the Bsrg I and BamHI restriction sites of the HIV-1 self-inactivating vector backbone pRRL-SIN18-cPPT-hPGK-EGFP-WPRE (pHIV-hPGK-GFPW+, [48]) under the control of the internal human phosphoglycerate kinase (hPGK) promoter. Retrovirus carrying the E1E2-HIV-SIN sequence was produced by co-transfection of the Gag pol packaging construct pCMVdeltaR8.91 [49] and glycoprotein expression construct pCMV-VSV-G (AJ 318514) in 293T cells (ATCC CRL-1573).

2.4×10^6^ 293T cells per 55 cm^2^ culture dish (Corning) were seeded 24 h prior to transfection in Dulbecco's modified Eagle's medium (DMEM) supplemented with 2 mM glutamine, 100 i.u./ml penicillin, 100 µg/ml streptomycin and 10% heat-inactivated FCS in a 95% humidified incubator containing 5% CO2 in air at 37°C. For the production of E1E2-HIV particles, 13.2 µg of pCMV-VSV-G envelope plasmid, 10.2 µg of pCMVdeltaR8.91 packaging plasmid and 13.2 µg of E1E2-HIV-SIN derived vector plasmid were co-transfected by calcium phosphate precipitation. Medium was replaced with 0.1 ml/cm^2^ of fresh culture medium without serum 14–16 h post-transfection.Viral supernatants were harvested 24 h later, cleared by low-speed centrifugation, and filtered through a 0.45 µm low binding protein filter (Millex filter unit, Millipore) prior to concentration. High-titer viral stocks were prepared by concentrating viral supernatants about 100 fold through ultracentrifugation for 2 h in a Beckman LE 70 ultracentrifuge at 26 000 rpm at 4°C in an SW28 rotor. Viral stocks were aliquoted in PBS 1% glycerol and stored at −80°C. 7 ng of p24 antigen (HIV P24 II Vidas, Vidas apparatus, BioMérieux) with 8 µg/ml polybrene in culture medium was used to infect overnight target cells, plated at 100 000 in a 2 cm^2^ well the day before.

### Cell culture

Caco-2 cells (ATCC HTB-37) were plated on inserts at a density of 250 000 cells /4.2 cm^2^ transwell and grown until confluency (∼6 days post seeding) in DMEM (Dulbecco's modified Eagle's medium) containing 25 mM glucose and 2 mM glutamine, supplemented with penicillin (100 i.u./ml) and streptomycin (100 µg/ml), 1% non-essential amino acids and 20%(v/v) heat-inactivated FCS. Cells were then cultured for an additional week under asymmetrical conditions with media containing FCS in the lower compartment and media without FCS in the upper compartment. Media were changed every 48 hours. For incubations with MTP inhibitor CP346086, fresh medium without FCS was added to the lower and upper compartment at the final concentrations of inhibitor for 24 or 48 hours. HepG2 (ATCC HB-8065), Huh-7.5 (from Charles Rice, Rockefeller University, New York, New York), Huh-7 cells (from John Mac Lauchlan, MRC, Glasgow, UK), and Hela (ATCC CCL-2) were plated in 9.5 cm^2^ wells at a density of 400 000 cells/well and grown for 24 h or 48 hours (until 80% confluence) in DMEM containing 25 mM glucose and 2 mM glutamine, supplemented with penicillin (100 i.u./ml), streptomycin (100 µg/ml), 1% non-essential amino acids and 10% (v/v) heat-inactivated FCS before the following experiments. For experiments with the MTP inhibitor CP346086, fresh medium without FCS was added to the 9.5 cm^2^ wells containing 0, 1 or 10 nM inhibitor for 48 hours.

### Immunofluorescence

Cells were fixed with formaldehyde 4% in PBS (formaldehyde 37%, Merck) for 30 minutes at 4°C and permeabilized by triton X100 0.5% in PBS for 30 minutes at 4°C. Intracellular expression of HCV E2 protein was assayed by indirect immunofluorescence with anti-E2 H52 antibody (10 µg/ml) and Alexa Fluor 546 F(ab')_2_ fragment of goat anti-mouse immunoglobulin G (1 µg/ml, Invitrogen). Cells were countercolored with Hoechst 33 342 (0,2 µg/ml) and observed with a Leica DMIRB microscope.

### ApoB ELISA

ApoB concentrations in the medium of the lower compartment were determined by ELISA. Ninety-six-well flat-bottomed ELISA plates (Maxisorb; Nunc) were coated overnight at 4°C with 100 µl monoclonal anti-human apoB antibody (5 µg/ml; clone 1609) in PBS and then saturated with 2% BSA for 1 h. Samples were distributed at 100 µl per well. After 2 h incubation at 37°C and washing with PBS/ 0,05% Tween 20, 100 µl of peroxidase-conjugated goat anti-human apoB antibody (1.6 µg/ml) 100 µl per well in PBS/ 0.2% BSA were added for 90 min at 37°C. The plates were washed and o-phenylenediamine substrate was added (100 µl per well). The reaction was revealed for 30 min and absorbance was read at 450 nm. Standard curves were established with apoB dilutions ranging from 10 to 200 ng/ml apoB (ApoB kit, SFRI Diagnostics).

To normalized quantity of apoB produced by 1000 cells, the number of viable cells in 0.3 cm^2^ transwells was determined by CellTiter 96® AQueous Non-Radioactive Cell Proliferation Assay (Promega). The MTS conversion into formazan was realized into transwells according to manufacturer recommandations. The absorbance at 490 nm of formazan product in transwells supernatants was measured after transfer to a 96-well plate.

### Iodixanol density gradients

Iodixanol gradients were prepared as described by Nielsen et al. [18]. Isopycnic linear density gradients were prepared from 6% (wt/vol) (1.7 ml of 60% [wt/vol] iodixanol, 0.34 ml of 0.5 M Tris-HCl, pH 8.0, 0.34 ml of 0.1 M EDTA, pH 8.0, and 14.6 ml 0.25 M sucrose) and 56.4% (wt/vol) (16.0 ml of 60% iodixanol, 0.34 ml of 0.5 M Tris-HCl, pH 8.0, 0.34 ml of 0.1 M EDTA, pH 8.0, and 0.34 ml 0.25 M sucrose) iodixanol solutions in thinwall centrifuge tubes (14×89 mm, Beckman) using a two-chamber gradient maker. A sample of 1 ml lower compartment medium was applied to the top of 6 to 56% iodixanol gradients and centrifuged for 10 h in a Beckman Optima L100 XP ultracentrifuge at 41000 rpm and 4°C in an SW41 rotor. The gradient was harvested by tube puncture from the bottom and collected into 11 (1 ml each) or 22 fractions (0.5 ml each). The density of each fraction was determined by measuring the mass of 100 µL.

### Co-immunoprecipitation

Co-immunoprecipitations were performed with µMACS Protein G Magnetic MicroBeads according to the manufacturer's instructions (Miltenyi Biotec, Bergisch Gladbach, Germany). 0.2 µg of anti-E2 H48 was added to 2 ml of the lower compartment medium before overnight incubation at 4°C. After one hour incubation with 20 µl of protein G microbeads, labelled immune-complexes were applied into a µMACS MS column. After one wash with PBS/BSA 0.2%, the retained immunoprecipitate was eluted in 100 µl of pre-heated (95°C) gel loading buffer.

### Western blot

Samples preparation: Media or gradient fractions were collected in Laemmli buffer and immediately frozen at −20°C until analysis. Cell layers were briefly rinsed twice with ice-cold PBS (Invitrogen), scraped into 0.5 ml of lysis buffer (1% Triton X-100 and 5 mM EDTA in PBS) supplemented with 2% (v/v) protease inhibitor cocktail (P8340; Sigma–Aldrich), and immediately frozen at −20°C. Cell lysates were run through a small bore needle (25G 5/8″) a few times. The protein concentration was determined using the Micro BCA Protein Assay (Perbio Science) with BSA as standard protein and samples were then diluted in loading buffer prior to fractionation by SDS-PAGE. Positive controls for ApoB100 and ApoB48 were obtained from purified human lipoproteins as follows. Plasma sample from a postprandial healthy donor was centrifuged for 4 h at 4°C and 543000 g with a TL100 ultracentrifuge (Beckman instruments). Upper low-density fraction was collected and 100-fold diluted in Laemmli buffer before denaturation. Positive controls for E1 and E2 were obtained from a lysate of cells expressing HCV glycoproteins.

20 µg of cell protein lysates, 40 µl of gradient fraction or 20 µl of immunoprecipitate were heated for 5 minutes at 95°C, fractionated by SDS-PAGE and transferred to a PVDF membrane. After an overnight incubation at 4°C in PBS-0.1% Tween 20 supplemented with 5% (w/v) non-fat milk powder, blots were probed with mAb against apoB, E1 or E2 diluted in PBS-0.1% Tween 20 (1∶5000 for apoB, 1∶1000 for E1 and E2 ) for 90 minutes followed by a peroxidase-conjugated goat anti-mouse antibody (Perbio Science; 1∶5000 dilution for 1 h in the same buffer). Blots were developed with enhanced chemiluminescence reagents according to the manufacturer's instructions (SuperSignal West Femto Maximum Sensitivity Substrate , Perbio Science).

### MTP Activity

MTP activity was measured with the Research Assay Kit for MTP (Chylos Inc.) according to manufacturer instructions with minor modifications. Briefly, for kinetic study, cells in inserts were washed with ice-cold PBS, dryed and frozen at day 7, 9, 11 and 13, and stored at −20°C until MTP activity assay. Cells from a 4.2 cm^2^ insert were homogenized into 150 µl of 50 mM tris-HCl, pH 7.4, 50 mM KCl and 5 mM EDTA homogenization buffer. Cell lysates were run through a small bore needle (25G 5/8″) twenty times. The assay was done in white microplates (Nunc GmbH, Wiesbaden, Germany). To the wells, we added 10 µl of cell homogenate, 5 µl of substrate vesicles mix and distilled water to make a final volume of 100 µl. Microplates were incubated at 37°C for 2 h before fluorescence reading using 460 nm excitation and 530 nm emission wavelengths into a Wallac 1420 Victor2™ microplate Fluorimeter (PerkinElmer, Courtaboeuf, France). To determine background fluorescence value, vesicles fluorescence was measured in absence of cell lysate after 2 h incubation in the same conditions. Background value was then subtracted from each sample. Protein concentrations in cell homogenates were determined using a Bradford protein assay kit (Sigma-Aldrich) and results were expressed as units of fluorescence per µg of protein.

## References

[1] Choo QL, Kuo G, Weiner AJ, Overby LR, Bradley DW (1989). Isolation of a cDNA clone derived from a blood-borne non-A, non-B viral hepatitis genome.. Science.

[2] Robertson B, Myers G, Howard C, Brettin T, Bukh J (1998). Classification, nomenclature, and database development for hepatitis C virus (HCV) and related viruses: proposals for standardization. International Committee on Virus Taxonomy.. Arch Virol.

[3] Appel N, Schaller T, Penin F, Bartenschlager R (2006). From structure to function: new insights into hepatitis C virus RNA replication.. J Biol Chem.

[4] Moradpour D, Penin F, Rice CM (2007). Replication of hepatitis C virus.. Nat Rev Microbiol.

[5] Rey FA (2003). Dengue virus envelope glycoprotein structure: new insight into its interactions during viral entry.. Proc Natl Acad Sci U S A.

[6] Bressanelli S, Stiasny K, Allison SL, Stura EA, Duquerroy S (2004). Structure of a flavivirus envelope glycoprotein in its low-pH-induced membrane fusion conformation.. Embo J.

[7] Mukhopadhyay S, Kim BS, Chipman PR, Rossmann MG, Kuhn RJ (2003). Structure of West Nile virus.. Science.

[8] Thomssen R, Bonk S, Propfe C, Heermann KH, Kochel HG (1992). Association of hepatitis C virus in human sera with beta-lipoprotein.. Med Microbiol Immunol (Berl).

[9] Thomssen R, Bonk S, Thiele A (1993). Density heterogeneities of hepatitis C virus in human sera due to the binding of beta-lipoproteins and immunoglobulins.. Med Microbiol Immunol (Berl).

[10] Andre P, Komurian-Pradel F, Deforges S, Perret M, Berland JL (2002). Characterization of low- and very-low-density hepatitis C virus RNA-containing particles.. J Virol.

[11] Lindenbach BD, Meuleman P, Ploss A, Vanwolleghem T, Syder AJ (2006). Cell culture-grown hepatitis C virus is infectious in vivo and can be recultured in vitro.. Proc Natl Acad Sci U S A.

[12] Maillard P, Krawczynski K, Nitkiewicz J, Bronnert C, Sidorkiewicz M (2001). Nonenveloped nucleocapsids of hepatitis C virus in the serum of infected patients.. J Virol.

[13] Lindenbach BD, Evans MJ, Syder AJ, Wolk B, Tellinghuisen TL (2005). Complete Replication of Hepatitis C Virus in Cell Culture.. Science.

[14] Wakita T, Pietschmann T, Kato T, Date T, Miyamoto M (2005). Production of infectious hepatitis C virus in tissue culture from a cloned viral genome.. Nat Med.

[15] Zhong J, Gastaminza P, Cheng G, Kapadia S, Kato T (2005). Robust hepatitis C virus infection in vitro.. Proc Natl Acad Sci U S A.

[16] Diaz O, Delers F, Maynard M, Demignot S, Zoulim F (2006). Preferential association of hepatitis C virus with apolipoprotein B48-containing lipoproteins.. J Gen Virol.

[17] Nielsen SU, Bassendine MF, Burt AD, Bevitt DJ, Toms GL (2004). Characterization of the genome and structural proteins of hepatitis C virus resolved from infected human liver.. J Gen Virol.

[18] Nielsen SU, Bassendine MF, Burt AD, Martin C, Pumeechockchai W (2006). Association between hepatitis C virus and very-low-density lipoprotein (VLDL)/LDL analyzed in iodixanol density gradients.. J Virol.

[19] Pumeechockchai W, Bevitt D, Agarwal K, Petropoulou T, Langer BC (2002). Hepatitis C virus particles of different density in the blood of chronically infected immunocompetent and immunodeficient patients: Implications for virus clearance by antibody.. J Med Virol.

[20] Bradley D, McCaustland K, Krawczynski K, Spelbring J, Humphrey C (1991). Hepatitis C virus: buoyant density of the factor VIII-derived isolate in sucrose.. J Med Virol.

[21] Hijikata M, Shimizu YK, Kato H, Iwamoto A, Shih JW (1993). Equilibrium centrifugation studies of hepatitis C virus: evidence for circulating immune complexes.. J Virol.

[22] Diaz O, Cubero M, Trabaud MA, Quer J, Icard V (2008). Transmission of low-density hepatitis C viral particles during sexually transmitted acute resolving infection.. J Med Virol.

[23] Hussain MM, Kancha RK, Zhou Z, Luchoomun J, Zu H (1996). Chylomicron assembly and catabolism: role of apolipoproteins and receptors.. Biochim Biophys Acta.

[24] Deforges S, Evlashev A, Perret M, Sodoyer M, Pouzol S (2004). Expression of hepatitis C virus proteins in epithelial intestinal cells in vivo.. Journal of General Virology.

[25] Andre P, Perlemuter G, Budkowska A, Brechot C, Lotteau V (2005). Hepatitis C virus particles and lipoprotein metabolism.. Semin Liver Dis.

[26] Nielsen SU, Bassendine MF, Martin C, Lowther D, Purcell PJ (2008). Characterization of hepatitis C RNA-containing particles from human liver by density and size.. J Gen Virol.

[27] Chang KS, Jiang J, Cai Z, Luo G (2007). Human apolipoprotein e is required for infectivity and production of hepatitis C virus in cell culture.. J Virol.

[28] Gastaminza P, Cheng G, Wieland S, Zhong J, Liao W (2008). Cellular determinants of hepatitis C virus assembly, maturation, degradation, and secretion.. J Virol.

[29] Huang H, Sun F, Owen DM, Li W, Chen Y (2007). Hepatitis C virus production by human hepatocytes dependent on assembly and secretion of very low-density lipoproteins.. Proc Natl Acad Sci U S A.

[30] Dubuisson J, Penin F, Moradpour D (2002). Interaction of hepatitis C virus proteins with host cell membranes and lipids.. Trends Cell Biol.

[31] Lavie M, Goffard A, Dubuisson J (2007). Assembly of a functional HCV glycoprotein heterodimer.. Curr Issues Mol Biol.

[32] Chateau D, Pauquai T, Delers F, Rousset M, Chambaz J (2005). Lipid micelles stimulate the secretion of triglyceride-enriched apolipoprotein B48-containing lipoproteins by Caco-2 cells.. J Cell Physiol.

[33] Delie F, Rubas W (1997). A human colonic cell line sharing similarities with enterocytes as a model to examine oral absorption: advantages and limitations of the Caco-2 model.. Crit Rev Ther Drug Carrier Syst.

[34] Bartosch B, Ciczora Y, Montigny C, Verney G, Wychowski C (2007). E1 envelope glycoprotein from hepatitis C virus particles exists as a transmembrane domain-dependent trimer..

[35] Chandler CE, Wilder DE, Pettini JL, Savoy YE, Petras SF (2003). CP-346086: an MTP inhibitor that lowers plasma cholesterol and triglycerides in experimental animals and in humans.. J Lipid Res.

[36] Perlemuter G, Sabile A, Letteron P, Vona G, Topilco A (2002). Hepatitis C virus core protein inhibits microsomal triglyceride transfer protein activity and very low density lipoprotein secretion: a model of viral-related steatosis.. Faseb J.

[37] Mirandola S, Realdon S, Iqbal J, Gerotto M, Dal Pero F (2006). Liver microsomal triglyceride transfer protein is involved in hepatitis C liver steatosis.. Gastroenterology.

[38] Domitrovich AM, Felmlee DJ, Siddiqui A (2005). Hepatitis C virus nonstructural proteins inhibit apolipoprotein B100 secretion.. J Biol Chem.

[39] Fisher EA, Ginsberg HN (2002). Complexity in the secretory pathway: the assembly and secretion of apolipoprotein B-containing lipoproteins.. J Biol Chem.

[40] Hussain MM, Shi J, Dreizen P (2003). Microsomal triglyceride transfer protein and its role in apoB-lipoprotein assembly.. J Lipid Res.

[41] Shelness GS, Sellers JA (2001). Very-low-density lipoprotein assembly and secretion.. Curr Opin Lipidol.

[42] Mensenkamp AR, Havekes LM, Romijn JA, Kuipers F (2001). Hepatic steatosis and very low density lipoprotein secretion: the involvement of apolipoprotein E.. J Hepatol.

[43] Mensenkamp AR, Teusink B, Baller JF, Wolters H, Havinga R (2001). Mice expressing only the mutant APOE3Leiden gene show impaired VLDL secretion.. Arterioscler Thromb Vasc Biol.

[44] Buhman KK, Accad M, Novak S, Choi RS, Wong JS (2000). Resistance to diet-induced hypercholesterolemia and gallstone formation in ACAT2-deficient mice.. Nat Med.

[45] Smith SJ, Cases S, Jensen DR, Chen HC, Sande E (2000). Obesity resistance and multiple mechanisms of triglyceride synthesis in mice lacking Dgat.. Nat Genet.

[46] Miyanari Y, Atsuzawa K, Usuda N, Watashi K, Hishiki T (2007). The lipid droplet is an important organelle for hepatitis C virus production.. Nat Cell Biol.

[47] Shavinskaya A, Boulant S, Penin F, McLauchlan J, Bartenschlager R (2007). The Lipid Droplet Binding Domain of Hepatitis C Virus Core Protein Is a Major Determinant for Efficient Virus Assembly.. J Biol Chem.

[48] Dupuy FP, Mouly E, Mesel-Lemoine M, Morel C, Abriol J (2005). Lentiviral transduction of human hematopoietic cells by HIV-1- and SIV-based vectors containing a bicistronic cassette driven by various internal promoters.. J Gene Med.

[49] Zufferey R, Nagy D, Mandel RJ, Naldini L, Trono D (1997). Multiply attenuated lentiviral vector achieves efficient gene delivery in vivo.. Nat Biotechnol.

